# Oxidative Stress in Ischemic Heart Disease

**DOI:** 10.1155/2020/6627144

**Published:** 2020-12-29

**Authors:** Aleksandar Kibel, Ana Marija Lukinac, Vedran Dambic, Iva Juric, Kristina Selthofer-Relatic

**Affiliations:** ^1^Department for Heart and Vascular Diseases, Osijek University Hospital, Osijek, Croatia; ^2^Department of Physiology and Immunology, Faculty of Medicine, University J.J. Strossmayer in Osijek, Osijek, Croatia; ^3^Department of Rheumatology and Clinical Immunology, Osijek University Hospital, Osijek, Croatia; ^4^Faculty of Medicine, University J.J. Strossmayer in Osijek, Osijek, Croatia; ^5^Department for Emergency Medical Services of the Osijek-Baranja county, Osijek, Croatia; ^6^Department of Internal Medicine, Faculty of Medicine, University J.J. Strossmayer in Osijek, Osijek, Croatia

## Abstract

One of the novel interesting topics in the study of cardiovascular disease is the role of the oxidation system, since inflammation and oxidative stress are known to lead to cardiovascular diseases, their progression and complications. During decades of research, many complex interactions between agents of oxidative stress, oxidation, and antioxidant systems have been elucidated, and numerous important pathophysiological links to na number of disorders and diseases have been established. This review article will present the most relevant knowledge linking oxidative stress to vascular dysfunction and disease. The review will focus on the role of oxidative stress in endotheleial dysfunction, atherosclerosis, and other pathogenetic processes and mechanisms that contribute to the development of ischemic heart disease.

## 1. Introduction

Atherosclerosis is the most common form of large vessel pathology responsible for syndromes of vital organ ischemic damage like myocardial infarction [1].

The key pathophysiologic process of atherosclerosis is chronic inflammation, where oxidative stress plays an essential role in vascular homeostasis regulation including endothelial and smooth muscle cell growth, proliferation, and migration; angiogenesis; apoptosis; vascular tone; host defenses; and genomic stability. Imbalance in the oxidant/antioxidant mechanisms leads to oxidative stress and uncontrolled vascular injury [2–4].

The relation between heart failure and vascular disease is also marked by oxidative stress, caused by ischemia, left ventricular (LV) dysfunction, and neuroendocrinological activation. Reactive oxygen species (ROS) negatively affect myocardial calcium handling, cause arrhythmias, and contribute to cardiac remodeling by inducing hypertrophic signaling, apoptosis, and necrosis. Neurohumoral activation via the renin-angiotensin-aldosterone system (RAAS) and the sympathetic nervous system (SNS), combined with increased pre- and after-load, impose additional myocardial oxidative stress [5].

Ageing, traditional cardiovascular risk factors (arterial hypertension, dyslipidemia, diabetes mellitus and smoking), genetic predisposition, and environmental factors increase ROS generation and decrease endothelial nitric oxide (NO) production. Additional factors like mechanic vascular properties and geometry, hemodynamic forces, and endothelial gene regulation by biomechanical forces (atheroprone and atheroprotective phenotypes), disturbed flow in vascular regions like arches, branches, and bifurcations can promote vascular injury, ROS activity, coronary atherosclerosis, and ischemic heart failure development [6, 7]. The gut microbiota is involved in mediating metabolic processes associated with risk factors for coronary artery disease such as obesity, dyslipidemia, diabetes mellitus, and dyslipidemia. These comorbidities via its metabolites can induce development of atheroslerosis and aterosclerotic coronary artery disease. The main pathways for these processes are provided via oxidative stress, inflammation, cholesterol, and uric acid metabolism [8, 9].

The current therapeutic approach for atherosclerotic vascular plaque stabilization and disease includes RAAS inhibitors, statins, and acetylsalicylic acid, because of their pleiotropic antioxidative effects [10–12]. There is a need to elucidate oxidative stress physiology and pathophysiology, to identify novel therapeutic modalities for selective oxidative stress targeting in atherosclerosis [4].

## 2. Ischemic Heart Disease

**Figure 1:**
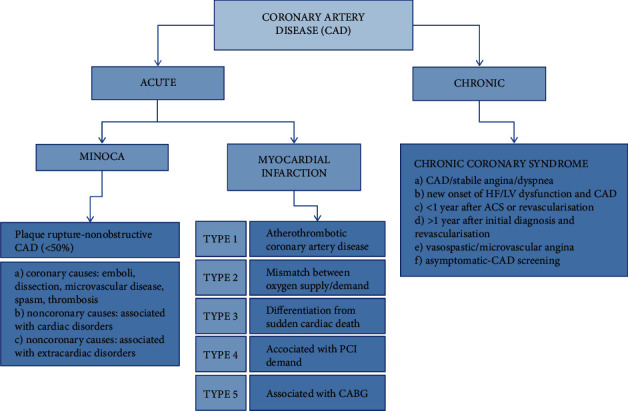
Acute and chronic coronary syndrome definitions and classification [14, 17].

Myocardial infarction (MI) is defined by clinical presentation, new ischemic electrocardiogram changes, and cardiac biomarkers elevation. The cause of MI is acute myocardial injury. Prolonged ischemia (a restriction in tissue blood supply, causing a deficiency of oxygen) can lead to myocardial necrosis and cell death. According to the Fourth Universal Definition of Myocardial Infarction 2018, MI can be divided into five categories (Figure 1).

MI type 1—caused by atherothrombotic coronary artery disease (CAD) and usually precipitated by atherosclerotic plaque disruption (rupture or erosion)

MI type 2—result of a mismatch between oxygen supply and demand: (a) reduced myocardial perfusion—coronary artery spasm, microvascular dysfunction, coronary embolism, coronary artery dissection, sustained bradyarrhythmia, hypotension or shock, respiratory failure, and severe anemia; (b) increased myocardial oxygen demand—sustained tachyarrhythmia and severe hypertension with or without LV hypertrophy

MI type 3—differentiation from sudden cardiac death

MI type 4—associated with percutaneous coronary intervention (PCI)

MI type 5—associated with coronary artery bypass grafting [7]

MINOCA (myocardial infarction with nonobstructive coronary arteries) can cause MI presenting with typical symptoms for acute coronary syndrome (ACS) and ST-segment elevation or equivalent. The underlying cause of disease may be nonobstructive (<50%) coronary artery disease stenosis, or mismatch between oxygen supply and demand, or secondary to myocardial disorders without involvement of the coronary arteries as myocarditis or Takotsubo syndrome [13].

Coronary artery disease (CAD) is a chronic, mostly progressive pathological process with predominant serious prognosis. This process can be modified by conservative and invasive treatment to achieve disease stabilization or regression [14]. Other cardiac conditions that are related to secondary myocardial injury are heart failure, myocarditis, any type of cardiomyopathy, Takotsubo syndrome, coronary revascularization procedure, cardiac procedure other than revascularization, catheter ablation, defibrillator shocks, cardiac contusion, systemic conditions, sepsis, infectious disease, chronic kidney disease, stroke, subarachnoid hemorrhage, pulmonary embolism, pulmonary hypertension, infiltrative diseases, amyloidosis, sarcoidosis, chemotherapeutic agents, critically ill patients, and strenuous exercise [15].

The leading symptom that initiates the diagnostic and therapeutic cascade in patients with suspected ACS is chest pain. Two groups of patients should be differentiated based on the electrocardiogram (ECG): those with persistent ST-segment elevation and those without persistent ST-segment elevation (transient ST-segment elevation, persistent or transient ST-segment depression, T wave inversion, flat T waves or pseudonormalization of T waves, or with normal ECG). The pathological finding at the myocardial level is cardiomyocyte necrosis or myocardial ischemia without cell loss [16].

Criteria for type 1 MI and type 2 MI detection are rise and/or fall of upper reference limit (URL) values with at least one value above the 99th percentile URL, with at least one of the following criteria: acute myocardial ischemia symptoms, new ischemic ECG changes, pathological Q wave development, and imaging evidence of new loss of viable myocardium or new regional wall motion abnormality in a pattern consistent with an ischemic etiology. For type 1 MI identification of a coronary thrombus by angiography including intracoronary imaging or by autopsy is needed as a one of the criteria, while evidence of an imbalance between myocardial oxygen supply and demand unrelated to acute coronary atherothrombosis for type 2 MI is a part of key definition [15].

The dynamic nature of the CAD results in various clinical presentations, which can be categorized as acute and chronic coronary syndromes. The diagnostic approach and management for patients with dyspnea and suspected ACS include assessment of symptoms and signs of disease, evaluation of the patient's general condition and quality of life, comorbidities evaluation, basic testing and assessment of LV function, risk assessment of obstructive CAD, diagnostic testing for CAD, and further event risk determination and treatment [14].

Management—including diagnosis and treatment—of acute ACS starts from the point of first medical contact. Out- and in-hospital treatment in acute setting is obligatory: relief of pain, breathlessness, and anxiety; arrhythmia management; reperfusion with PCI alone or/with fibrinolysis strategy, and periprocedural pharmacologic and nonpharmacologic therapy in a coronary unit [13].

Long-term management following acute treatment includes life style intervention, risk factor control, blood pressure and dyslipidemia treatment, glucose lowering therapy, antithrombotic therapy in acute and long-term settings, possible heart failure treatment, and arrhythmia management. Cardiac rehabilitation should be recommended [13].

CAD is a chronic, progressive disease with a predominantly serious prognosis. The outcome of MINOCA strongly depends on the underlying cause, and its overall prognosis is serious, with a 1-year mortality of about 3.5% [13, 14].

## 3. Endothelial Dysfunction and Oxidative Stress

Endothelial dysfunction caused by oxidative stress is an early event in the pathogenesis of many cardiovascular diseases including atherosclerosis, dyslipidemia, hypertension, diabetes, chronic kidney disease, heart failure, and ischaemia/reperfusion injury [18–23], and it is a hallmark of vascular diseases. An imbalance between NO bioavailability and ROS, also called oxidative stress, promotes endothelial dysfunction [24, 25] which is characterized by an altered modulation of vasomotor tone and vascular growth, impaired anti-inflammatory and antithrombotic endothelial characteristics, and disturbances of vascular remodeling [26].

The endothelium is a simple squamous layer of cells that forms an interface between the circulating blood and the vascular wall. A healthy endothelium provides endothelium-dependent vasorelaxation in response to vascular stress, controls vascular permeability, and prevents platelet aggregation [27]. It is very reactive to mechanical stimuli, chemical factors, and humoral agents by producing several mediators, such as NO, to maintain vasomotor tone and structural integrity. NO has a major role in endogenous antioxidant defense because of its potent vasodilatory, anti-inflammatory, and antithrombotic characteristics [28, 29]. Most of the vascular NO is produced by endothelial nitric oxide synthase (eNOS), a cytochrome p450 reductase-like enzyme which uses tetrahydrobiopterin to form NO from *L*-arginine [19]. The main causes of reduced NO bioavailability include increased NO degradation caused by ROS, decreased expression of eNOS, deficiency of substrates or cofactors for eNOS, and an inappropriate activation of eNOS caused by impaired cellular signaling [19, 30, 31]. Also, previous studies examined the phenomenon called eNOS uncoupling, causing reduced NO bioavailability by eNOS switching its enzymatic activity to generate superoxide (O_2_^−^) and H_2_O_2_ instead of NO [32, 33]. This occurs, for example, in the absence of NOS substrate *L*-arginine or the cofactor tetrahydrobiopterin in that process [34, 35]. Besides eNOS, which is mostly expressed in endothelial cells, there are two more isoforms of NO synthase with other functions—neuronal NOS (nNOS) and inducible NOS (iNOS) [36], which can also be a subject to uncoupling [33].

ROS are the products of the normal cellular aerobic metabolism generated during the reduction of oxygen [19]. ROS include unstable free radicals such as superoxide anion (O_2_^−^), hydroxyl radical or lipid radicals, and nonfree radicals such as hydrogen peroxide (H_2_O_2_), hypoclorous acid, or peroxynitrite which also have oxidizing effects that contribute to oxidative stress [19]. At moderate concentrations, ROS exert some physiological roles such as signaling [19, 37], but increased production of ROS which exceeds endogenous antioxidant defense mechanisms causes oxidizing of DNA, proteins, carbohydrates, lipids, and other biological macromolecules, leading to oxidative stress [6]. Enzymatic sources of ROS that are important in the cardiovascular system are NADPH (reduced form of nicotinamide adenine dinucleotide phosphate) oxidase, xanthine oxidase, and uncoupled eNOS with an addition of the mitochondrial electron transport chain, cyclooxygenase, and lipoxygenase as additional possible sources [6, 19, 32, 37]. Furthermore, production of ROS may be enhanced by free radical chain reactions. Several studies showed a very important role of NADPH oxidase, including Nox family oxidases Nox1, Nox2, Nox4, and Nox5, in endothelial dysfunction [19, 32]. NADPH oxidase is an enzyme located in the membrane of endothelial cells, smooth muscle cells, and fibroblasts, and it is the most powerful source of O_2_^−^ production [38]. Angiotensin II, thrombin, platelet-derived growth factor, tumor growth factor-*α*, and lactosylceramide upregulate this enzyme and cause excessive ROS production [19]. Previous studies regarding angiotensin II-induced hypertension, diabetes mellitus, and hypercholesterolemia demonstrated the important impact of NADPH oxidase [23, 38, 39]. Xanthine oxidase is an enzyme that has a role in oxidation of hypoxanthine and xanthine in the metabolism of purines, leading to production of O_2_^−^ and H_2_O_2_. The activity and expression of this enzyme are increased by interferon-*γ* [19]. The role of xanthine oxidase in ROS production in hypertension and hypercholesterolemia has been discovered in previous research [40–42]. As mentioned before, all three isoforms of NOS can be a source of ROS when uncoupling occurs, and NOS starts producing O_2_^−^ and H_2_O_2_ instead of NO, but uncoupled eNOS products play a critical role in the pathogenetic processes of cardiovascular diseases [33]. This was shown in previous studies regarding hypertension, hypercholesterolemia, smoking, and diabetes mellitus [6, 43, 44]. Mitochondrial oxidative phosphorylation normally produces physiological levels of superoxide, which is converted to hydrogen peroxide and afterwards to water. Mitochondrial oxidative stress can be a consequence of excessive ROS production or insufficient ROS detoxification [39].

**Figure 2:**
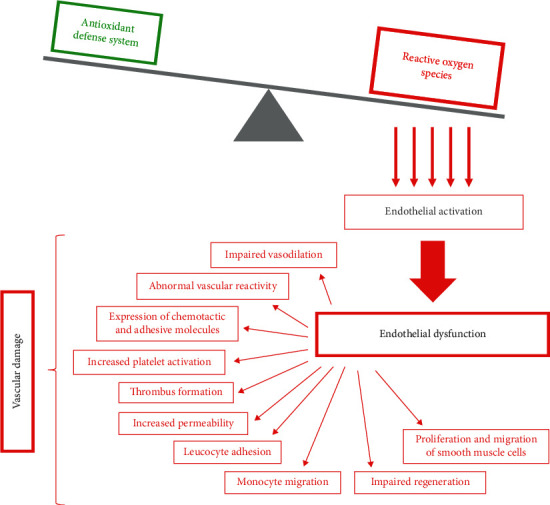
Endothelial dysfunction and development of vascular damage.

Excessive ROS production exceeding antioxidant defense systems leads to endothelial oxidative stress. The first step of endothelial dysfunction is called endothelial activation, which represents the expression of abnormal prothrombotic and proinflammatory characteristics of the endothelial cells, leading to other chronic changes [45]. Endothelial dysfunction includes impaired endothelium-mediated vasodilation; abnormal vascular reactivity and vasospasm; greater expression of chemotactic and adhesive molecules; increased platelet activation and thrombus formation; increased permeability of endothelium, leucocyte adhesion, and monocyte migration into the vascular wall; and impaired regeneration of endothelial cells with proliferation and migration of smooth muscle cells, leading to vascular damage [32, 46] (Figure 2).

Many studies have demonstrated an important role of oxidative stress in endothelial dysfunction under the conditions of excessive oxidative stress. Cardiovascular risk factors cause imbalances between NO and ROS, so prevention of endothelial dysfunction by reducing oxidative stress and enhancement of endothelial NO production is seen as a reasonable therapeutic strategy in cardiovascular diseases [6, 46].

## 4. Oxidative Stress in Atherosclerosis

Atherosclerosis is a multisystemic, progressive, chronic inflammatory disease characterized by the interaction of immune and endothelial cells that is mediated by adhesion molecules on the surface of the vascular endothelium leading to the release of numerous proinflammatory mediators [47]. Specifically, it has been demonstrated that there is a close interaction between vascular endothelial inflammation and intense oxidative stress in triggering the atherosclerotic process [48].

The imbalance between the generation of excess reactive oxygen species (ROS) and the antioxidant mechanism leads to increased oxidative stress resulting in the formation of atherogenic oxidized low-density lipoprotein (Ox-LDL) which is a major determinant of atherogenesis [49].

Production of ROS from various sources (xanthine oxidase, lipoxygenase, nicotinamide adenine dinucleotide phosphate oxidase, eNOS, nNOs, and iNOS) leads to damage to mitochondrial capacity and to the development of mitochondrial dysfunction [50]. Free fatty acids in endothelial cells enter the tricarboxylic acid cycle during which oxidation results in the overformation of NADH, which is an important driver of ROS during oxidative phosphorylation [51]. Mitochondrial dysfunction leads to increased ROS formation and oxidative stress and thus plays a role in the initiation, formation, and progression of an atherosclerotic lesion [51].

Studies have shown that increasing ROS production in mitochondria is induced by age, obesity, smoking, hypertension, diabetes, and dyslipidemia [52]. Numerous studies have found that mitochondrial dysfunction significantly affects the regulation of inflammation, proliferation, and apoptosis in the onset and progression of atherosclerotic plaques [53–57].

During the atherosclerotic process, the accumulated neutrophils produced an additional amount of ROS [58]. ROS enhance the activation of poly (ADPribose) polymerase 1 (PARP1), which damages mtDNA and thus the mitochondrial transport chain, further enhancing ROS formation and further damaging endothelial cells [59]. The resulting ROS trigger the synthesis of inflammatory cytokines by different cellular pathways resulting in vascular inflammation and participating in the oxidation process of LDL [58]. Ox-LDL has a cytotoxic effect on vascular cells, and macrophage removal receptors can phagocytose them, forming foaming cells that deposit in the blood vessel wall forming an atherosclerotic plaque [60]. Ox-LDL exerts its various effects on cells such as endothelial cells, macrophages, platelets, fibroblasts, and smooth muscle cells through transmembrane glycoproteins such as SR-A, CD36, and LOX-1 [61]. The resulting Ox-LDL increases the NADPH oxidase activity, leading to an increase in ROS synthesis and to NO inactivation. It also causes eNOS dysfunction by displacing it from the alveolar membrane site and enhances the arginase II activity thereby reducing the amount of *L*-arginine cosubstrate for eNOS resulting in an additional decrease in NO synthesis [62]. Ox-LDL increases the synthesis of matrix metalloproteinases (MMP), namely MMP-1, MMP-3, and MMP-9, leading to a breakdown of the fibrotic cap and to a consequent rupture of the atherosclerotic plaque. LOX-1 is expressed on macrophages, vascular endothelial smooth muscle cells, cardiomyocytes, platelets, and fibroblasts. The binding of Ox-LDL to LOX-1 in macrophages and vascular smooth muscle cells results in the formation of foam cells [63]. The main inducers of the LOX-1 expression are tumor necrosis factor-alpha (TNF-*α*), interleukin-1 (IL-1), interferon-gamma (IFN-*γ*), CRP, and modified lipoproteins such as glyxidized LDL, lysophosphatidylcholine, and ROS, while the mediators and conditions regulating the gene expression are numerous: angiotensin II, cytokines, glycation end products, diabetes mellitus, hypertension, dyslipidemia, ischemia reperfusion injury (IRI), heart failure, psychological stress, and HIV infection [61]. TNF-*α* and NF-kB increase Ca^2+^ levels in the mitochondria and consequently increase ROS production. SR-A and CD36 take up 75% to 90% of LDL [64]. SR-A is expressed in the presence of oxidative stress and growth factors in endothelial and smooth muscle cells, while normally found only in myeloid cells [65]. CD36 is found on monocytes, macrophages, platelets, and adipocytes [63]. Human macrophages lacking CD36 have a 40% reduction in Ox-LDL binding and uptake [66]. Toll-like receptors (TLRs) constitute a major subset of pattern recognition receptors (PRRs) that are significantly expressed on different immune cells during atherogenesis in the coronary circulation [67]. TLR signaling cascades can be activated by a wide range of endogenous ligands associated with tissue damage, which plays a central role in the development of atherosclerotic plaques [67]. The main culprits involved in the immune response to oxLDL are TLR4 [67]. Ox-LDL has been shown to lead to increase the expression of TLR4 in macrophages, neutrophils, and dendritic cells with which it plays an important role in the development of atherosclerotic plaques in the coronary circulation by activating MAPK and NF-*κ*B pathways [68, 69]. Activation of MAPK and NF-*κ*B transcription factors results in enhanced activation of genes encoding proinflammatory cytokines and chemokines important for the progression of the atherosclerotic process, including TNF-*α*, IL-1, and Il-6 [67]. miR-590 has antiapoptotic effects on endothelial cells attacked by the atherosclerotic process by inactivating the TLR4/NF-*κ*B pathway, which may be a potential therapeutic target [70]. TLR4 is required for Ox-LDL-induced differentiation of macrophages into foam cells in the early stages of atherosclerosis [71]. It plays a crucial role in plaque progression and rupture leading to occlusive thrombus formation in human coronary arteries [72]. Specific Ox-LDL derivatives act as TLR4 ligands by enhancing the MMP-9 expression [73]. Also, minimally modified low-density lipoproteins (mmLDL) via CD14 and TLR4 induce actin polymerization which together with MMP-9 leads to remodeling of the coronary artery wall, resulting in instability of atherosclerotic plaques and their rupture [74]. Cellular fibronectin (cFN) is an extracellular matrix protein (ECM) that is overexpressed only in chronically inflamed tissues and is synthesized by vascular smooth muscle cells and endothelial cells [75]. cFN activates macrophages and platelets via TLR4 resulting in platelet aggregation and arterial thrombosis within atherosclerotic lesions in the coronary arteries [75]. TLR2 activation stimulates VSMC migration from the intima in an IL-6-dependent manner, regulates inflammatory processes and ROS production after vascular injury, and contributes to coronary endothelial dysfunction after ischemic-reperfusion injury by activating neutrophils and creating ROS [67]. Ox-LDL can induce the expression of mRNAs of Wnt5a (Wnt family of glycoproteins) that are coexpressed with TLR2 and TLR4 and play a key role in the formation of foam cells, especially in advanced atherosclerotic plaques, which correlates with the severity of atherosclerotic lesions in human studies [76, 77]. TLR9 is expressed in the endoplasmic reticulum and not on cell surfaces such as TLR2 and TLR4 [67]. TLR9 is activated by CpG motifs in nucleic acids released during vascular necrosis and stimulates the transformation of macrophages into foam cells in a manner dependent on NF-*κ*B and IRF7 (interferon regulatory factor 7) and stimulates the secretion of INF and increases cytotoxic activity CD4 + T cell versus coronary artery smooth muscle cells [78].

VSMCs are important components of atherosclerotic plaques that, under the influence of biostimulation or mechanical damage triggered by oxidative stress, change their phenotype and, through differentiation, become synthetic VSMCs that produce significantly less contractile proteins, increase proliferation and migration, and thus participate in the development of atherosclerosis [79]. Increased concentrations of Ox-LDL via LOX-1 cause smooth muscle cell apoptosis as they increase the expression of a proapoptotic protein such as the bcl-2-associated X protein (Bax) leading to instability and rupture of the atherosclerotic plaque. In addition, through inducers, CD147 can cause plaque instability by releasing extracellular MMP [52].

Oxidative stress caused by the production of ROS and RNS (nitric oxide (NO), peroxynitrite (ONOO−)) and S-nitrosothiol (RSNO)) can damage macromolecules because it reacts with specific amino acid residues and DNA and chromatin cause mutations or double-stranded breaks in a phenomenon overall known as “oxidative damage” [80]. The selenoprotein family is involved in the control of oxidative stress in the cardiovascular system by inhibiting oxidative stress, modulating inflammation, suppressing endothelial dysfunction, and protecting vascular cells from apoptosis and calcification [81]. Potent selenoproteins of particular importance to the cardiovascular system are glutathione peroxidase (GPX), thioredoxin reductase 1 (TXNRD), methionine sulfoxide reductase B1 (MSRB1), selenoprotein P (SELENOP), selenoprotein S (SELENOS), and selenoprotein T (SELENOT) [81]. Dysfunction of various selenoproteins can lead to congestive heart failure, coronary heart disease, and to damaged heart structure and function [80]. The main catalytic site of selenoprotein is called Sec [80]. GPXs are the major components of the antioxidant system that maintain oxidative homeostasis, using glutathione as a cofactor for catalyzing the reduction of hydrogen peroxide (H2O2) and/or phospholipid hydroperoxide [80]. GPX3 controls vascular tone and the thrombotic properties of vascular endothelium [80]. TXNRD, along with thioredoxin (Trx) and NADPH, represents the major disulfide reduction system in the cell [82]. MSRB1 acts synergistically with GPX and TXNRD primarily in the liver, kidneys, and heart [80]. Selenoprteins P, S, and T predominantly contribute to calcium ion (Ca^2+^) signaling, protein folding, and ER-related degradation [80]. SELENOS, SELENOK, SELENOM, SELENON, SELENOF, and SELENOT are involved in maintaining the homeostasis of oxidative stress in the ER of cardiac myocytes [80]. Studies have shown that decreased selenoprotein levels are associated with the increased Nrf2 expression which may represent an important compensatory response to the maintenance of homeostasis [83]. Selenoproteins play an important role in embryogenesis, since it was found that mice that had a genetic disorder of cytosolic TXNRD1, mitochondrial TXNRD2, and GPX4 experienced embryonic mortality [84].

Polyunsaturated fatty acids (PUFAs) exert anti-inflammatory, antiatherogenic, and antioxidant properties on the cardiovascular system [85–87]. These important effects are achieved by competing with arachidonic acid (AA) for enzymes involved in the biosynthesis of proinflammatory mediator molecules, by suppressing proinflammatory NF-*κ*B by modulating TLR4 signaling, by activating PPAR-*γ*, and FFA4 receptors (before GPR120 d) in macrophages and metabolites such as esolvins, maresins, and protectins that have anti-inflammatory and antioxidant effects [88, 89]. The most studied molecular mechanisms are the activation of Nrf2 in the vascular tissue, leading to the production of antioxidant enzymes (HO-1, GPx) and the activation of FFA4 receptors, resulting in the preservation of *κ*B inhibitors (I*κ*B) and the prevention of NF-*κ*B nuclear translocation [90–93]. F2-isoprostanes are prostaglandin-like molecules formed as a result of peroxidation of ROS-mediated esterified arachidonic acid [94]. n-3 PUFAs reduce 8-isoprostane levels in macrophages and reduce oxidative stress [88]. The peroxidation products of *ω*-3 PUFAs and *ω*-6 PUFAs can also have toxic effects in oxidative stress, and a diet rich in PUFAs can lead to tissue hypersensitivity to lipid peroxidation induced by oxidative stress [95, 96]. Therefore, in the future, it will be necessary to investigate individually each potential PUFA that has been shown to be an important protective factor in oxidative stress.

**Figure 3:**
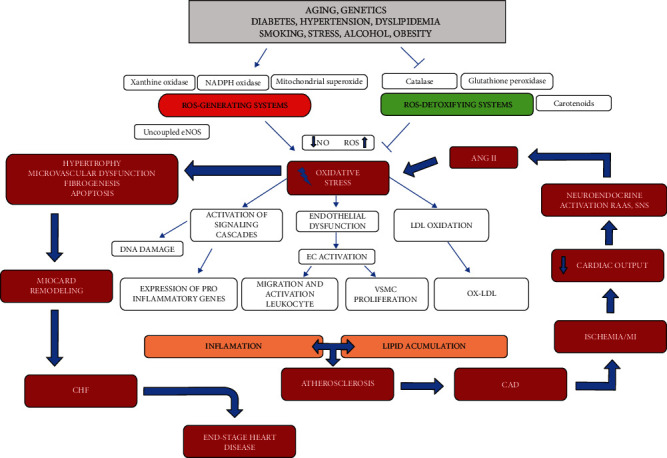
The role of oxidative stress in the onset of atherosclerosis and the pathophysiological implications of congestive heart failure. ANG II: angiotensin II; CAD: coronary artery disease; CHF: congestive heart failure; DNA: deoxyribonucleic acid; EC: endothelial cells; eNOS: endothelial nitric oxide synthase; LDL: low-density lipoprotein; MI: myocardial infarction; NO: nitric oxide; RAAS: renin–angiotensin*–*aldosterone system; ROS: reactive oxygen species; SNS: sympathetic nervous system; VSMC: vascular smooth muscle cells.

The most important antioxidants are glutathion peroxidase (Gpx), glutathion reductase, catalase, and superoxide dismutase [97]. Numerous studies have shown that efficient elimination of ROS from cells reduces the formation and progression of atherosclerotic plaques [98, 99]. The role of antioxidants on the progression of an atherosclerotic lesion needs to be further investigated as there are studies that confirm that certain antioxidants have no effect on the development of an atherosclerotic lesion [100]. A transcriptional coactivator that regulates a gene involved in energy metabolism in mitochondria called peroxisome proliferator-activated receptor gamma coactivator 1-alpha (PGC-1*α*) which is an important mitochondrial protector that promotes the synthesis of NO enzymes, mitochondrial protein 2 (UCP-2), and the antioxidant defense of mitochondria (manganese SOD, catalase, and thioredoxin 2), and this way limits endothelial dysfunction [52, 101]. PGC-1*α* also reduces the activity of the inflammatory factors NF-*κ*B and TNF-*α* and prevents the entry of Ox-LDL into cells [62, 102]. Twinkle mtDNA helicase plays a major role in stabilizing atheromatous plaques and reducing the development of atherosclerosis as it decreases apoptosis of VMSCs and macrophages [52]. Mitofusini 1 (Mfn1) is an important GTPase that regulates VMSC proliferation and apoptosis and acts as an important endogenous inhibitor of VSMC proliferation by inhibiting the Ras-Raf-ERK 1/2 pathway during the atherosclerotic process [103, 104]. Thus, prevention of vascular oxidative stress and improvement of NO production may be key future targets of new therapeutic strategies for the treatment of atherosclerosis [61].  Figure 3 summarizes the interactions of pathogenetic mechanisms linking oxidative stress to atherosclerosis, coronary artery disease, and consequently heart failure.

## 5. Oxidative Stress in Coronary Artery Disease

Dyslipidemia, as well as an imbalance between ROS production and enzymatic and nonenzymatic antioxidant protection systems, leads to endothelial dysfunction and atherosclerosis of the coronary arteries [1]. Numerous studies have shown impaired balance of prooxidants and antioxidants in patients with CAD [105–108]. Oxidative stress is today considered a new risk factor responsible for the development of CAD that affects the onset, prognosis, quality of life, and survival of patients [109].

In addition to being associated with atherosclerosis, oxidative stress can create oxidative modification or damage to lipid peroxidation at the level of deoxyribonucleic acids (DNA) and proteins with deleterious effects on the structure and function of the vascular system [110]. In addition to classical free oxygen radicals (superoxide radical (O2-), hydrogen peroxide (H2O2), hydroxyl (OH), peroxyl (RO), hydroperoxyl (HRO-)), reactive oxidative stress has also been shown to be involved in the oxidative stress process of nitrogen species (RNS), especially peroxynitrite (ONOO-) [111]. We know that ROS damages key molecules in signaling pathways involved in vascular inflammation, and it damages essential biomolecules in cells and participates in oxidative modification of lipids that make them atherogenic [112]. The most important sources of oxidative stress are the phagocytic isoform of NADPH oxidase (Nox2 and to a lesser extent Nox1) with its regulatory subunit p47phox, xanthine oxidase (XO), and dysregulated eNOS [113].

Common risk factors (hyperlipidemia, hyperglycemia, smoking, hypoxia, etc.) activate NADPH oxidase via different signaling pathways. It is now known that enhanced release of reactive oxygen species (ROS) by NADPH oxidases and mitochondrial enzymes results in cardiomyocyte hypertrophy, fibrosis, and an increase in metalloproteinase. The most studied mechanism of NADPH activation is mediated by one of the mechanisms of the PKC*α*/*β*2 signaling pathway in which protein kinase C plays a key role [114]. The p47phox subunit is the major Nox2 (gp91phox) regulatory subunit whose phosphorylation is required for Nox2 activation. The expression of p47phox was significantly increased in patients with CAD and overweight by about 60% and in obese patients with CAD by about 80%. So far, XO is known to be significantly elevated in CAD patients, and overweight is thought to be a potent driver of the enhanced XO expression [113].

In patients who have a BMI increase, suffer from CAD and will undergo CABG, increased ROS levels, increased expression of ROS-producing enzymes (P47phox, xanthine oxidase), decreased expression of antioxidant enzymes (mitochondrial aldehyde dehydrogenase, heme oxygenase-1, and eNOS), and increase in markers of inflammatory processes in serum and right atrial myocardial tissue (sVCAM-1 and CCL5/RANTES) have been demonstrated [115].

Endoplasmic reticulum stress (ERS) occurs in cardiac myocytes and cardiac tissue in response to various stressors, such as ischemia, hypoglycemia, hyperlipidemia, inflammation, and osmotic stress [116]. The resulting oxidative stress leads to changes in the redox status of the ER that interfere with the formation of protein disulfide and cause misfolding of the protein [116]. High cholesterol, fatty acids, and oxidative stress may induce ERS-induced apoptosis of macrophages and endothelial cells in atherosclerotic plaques [117]. ERS is associated with the development and progression of cardiac hypertrophy, ischemic heart disease, and heart failure [118]. The consequences of ERS are the accumulation of incorrectly posttranslationally modified secretory and transmembrane proteins that have important cellular functions [116]. During ERS, intracellular signaling pathways called *unfolded protein response* (UPR) are activated, restoring ER homeostasis, but if ERS persists chronically at high levels, terminal UPR activates cell apoptosis, which may be one of the important pathophysiological mechanisms for disease development [116]. Terminal UPR makes an important contribution to myocyte loss during myocardial infarction [119]. Also, it has been discovered that ER autophagy may be the last resort to restore ERS homeostasis [120]. There is also evidence that activation of UPR also activates Nrf2, which has been shown to be an important cardioprotective factor [121]. Improved understanding of the molecular mechanisms of regulated ERS in the future may lead to the discovery of new therapeutic targets [118].

ROS leads to the activation of the nuclear transcription factor kappa B (NF-*κ*B), which regulates key genes for the encoding of proinflammatory cytokines, chemokines, and leukocyte adhesion molecules. Two important transcription factors—nuclear factor erythroid 2-related factor 2 (Nrf2) and peroxisome proliferator-activated receptor-*β*/*δ* (PPAR*β*/*δ*)—have been shown to protect coronary blood vessels from excessive exposure to oxidative stress. Oxidative stress and inflammation are thought to be major activators of these protective transcription factors [122]. Nrf2 stimulates genes for the synthesis of antioxidant and detoxifying enzymes and indirectly antagonizes the proinflammatory effects of NF-*κ*B by removing ROS [123–125]. PPAR*β*/*δ* is predominantly located in the heart and has cardioprotective effects by suppressing the activity of several transcription factors, including NF-*κ*B [126].

Bone marrow endothelial progenitor cells (EPCs) are responsible for neovascularization and reendothelialization after ischemia and/or tissue injury, and a decrease in EPC numbers and their function has been demonstrated in CAD patients [114, 127]. High levels of oxidative stress in CAD patients are thought to be closely related to the enhanced activation of NDPH oxidase mediated by the membrane component p47phox, which plays a major role in the regulation of the NADPH activity and thus reduced vascular capacity of EPCs in CAD patients [114]. Medications used today, such as AT blockers, ACE inhibitors, statins, and tazolidindiones, have a beneficial effect on the bioactivity of EPCs that maintain vascular homeostasis [2]. During oxidative stress, serum EPC levels drop significantly, suggesting that this may serve as a good biomarker of oxidative stress [128].

Cytotoxic products of the enzyme myeloperoxidase (MPO), such as hypochlorous acid, lead to oxidative damage to blood vessels. Human MPO is an important pathophysiological mediator and biomarker in CAD patients whose levels are significantly elevated, leading to the formation of dysfunctional lipoproteins with increased atherogenic potential, decreasing NO availability, weakening vasoreactivity, and leading to atherosclerotic plaque instability [129]. Malonyldialdehyde (MDA) is one of the last products of peroxidation of polyunsaturated fatty acids in cells whose levels increase significantly during oxidative stress. Therefore, the level of human MDA in blood plasma is a very important biomarker of ROS-induced lipid peroxidation [130]. In a study on 30 patients with angiographically defined CAD and 30 healthy control subjects, serum MDA levels were increased, although these values did not differ depending on the number of affected coronary vessels and were not correlated with the severity of vascular lesions [131]. The level of MDA and the percentage of MDA release were significantly elevated, while the level of glutathione (GSH), erythrocyte GPx activity, and total plasma antioxidant capacity (TAC) was significantly reduced in patients with acute coronary syndrome and with CAD, compared to healthy subjects (*n* = 30/group) [112]. The study thus found that in patients with CAD, there was a significant decrease in glutathione in erythrocytes and consequently elevated levels and increased release of MDA, confirming that the susceptibility of erythrocyte membranes to oxidative stress was significantly higher in patients with CAD than in healthy subjects. Also, the same study showed results in which the erythrocyte level and total antioxidant capacity (TAC) value were significantly lower compared to healthy controls [112].

Various study groups have reported significant decreases in the parameters of antioxidants in patients with CAD. It is important to emphasize that, according to current data, patients with multivessel coronary artery stenoses have significantly higher levels of MDA and significantly lower levels of GSH, TAC, and GPx activity than patients with double and single coronary artery disease, which clearly leads to the conclusion that the greater the number of coronary artery stenoses, the higher the level of oxidative stress [112].

Abolhasani et al. conducted a study showing that the serum concentrations of high-sensitivity C-reactive protein (hs-CRP), sialic acid (SA), vitronectin (VN), plasminogen activator inhibitor-1 (PAI-1), Ox-LDL, and MDA were significantly elevated in patients with CAD relative to the healthy control group [132]. ROS-mediated lipid peroxidation leads to the formation of unsaturated aldehydes, including acrolein and MDA, which have toxic effects [112]. A study conducted by Yilmaz et al. showed that serum MDA was significantly higher, and TAC was significantly lower in CAD patients [133]. A study by Ninic et al. showed that the major lipid peroxidation product thiobarbiturate acid-reactive substances (TBARS) was significantly higher in patients with CAD than in the control group, while the antioxidant effect of many serum antioxidants was significantly lower [1]. TBARS leads to further formation of ROS and acts on proteins and DNA that exert proatherogenic and mutagenic effects [134]. Tumor necrosis factor-related apoptosis-inducing ligand (TRAIL) is a cytokine that acts as an apoptosis-inducing ligand, and research has shown that TRAIL levels are significantly reduced in animal CAD models, and that the unknown mechanism of TRAIL reduces oxidative stress and endothelial dysfunction [135].

Below, we present new insights into the numerous molecules, signaling pathways, and antioxidants involved in the highly complex development of oxidative stress in the coronary circulation. Iranian researchers have shown in a study that the increased expression of HSP27 mRNA in the peripheral blood mononuclear cell (PBMCs) is significantly associated with the severity of CAD and can serve as an important prognostic biomarker, indicating the degree of total oxidative stress [136]. A large study conducted by Khaper et al. showed that one week after acute myocardial infarction, the mRNA level for mitochondrial manganese superoxide dismutase (Mn SOD) decreased by 40% and after sixteen weeks by 73% compared with healthy subjects, an indicator of depleted antioxidant protection in patients with CAD [137].

In the animal model, the growth arrest-specific 5 (GAS5) overexpression in CAD rats has been shown to inhibit abnormal activation of the Wnt/*β*-catenin signaling pathway, leading to improvement of hyperlipidaemia, attenuation of myocardial injury, inhibition of cardiomyocyte apoptosis, and reduction of oxidative stress [138]. Decrease in leukocyte telomere length (TL) and mitochondrial DNA copy number (mtDNA-CN) are important indicators of the development of CAD, which are involved in the modulation of oxidative stress as independent risk factors, but this needs further investigation [139]. Polymorphisms in NRF2 and its target antioxidant genes: HMOX-1, NQO1, and MT significantly influence the level of oxidative stress in CAD formation [51, 140]. Inhibition of SAH hydrolase (SAHH) adenosine dialdehyde inhibitor in CAD patients leads to a significant increase in plasma S-adenosylhomocysteine (SAH) that promotes the production of free oxygen radicals and leads to endothelial dysfunction by epigenetic regulation of the oxidative stress pathway mediated by the p66shc gene promoter expression [141].

An antioxidant and an important component of the electron transport chain, coenzyme Q10 (CoQ10), has an effect on biomarkers of inflammation and oxidative stress, and the study found that CoQ10 significantly increased SOD and catalase (CAT) levels in CAD patients, significantly reduced MDA and dienes, and had significant effect on C-reactive protein (CRP), tumor necrosis factor-alpha (TNF-*α*), interleukin-6 (IL-6), and GPx levels [142]. Supplementation with L-carnitine at a dose of 1000 mg/d after 12 weeks reduces oxidative stress (MDA level by 7%) and increases the activity of antioxidant enzymes (CAT by 16%, SOD by 47%, and GPx by 12%) in patients with CAD [143], while the administration of doses higher than 2000 mg/d showed a cardioprotective effect and reduced mortality rates in CAD patients [144].

Protein phosphatase and actin regulator 1 (PHACTR1), which regulates the reorganization of the actin cytoskeleton, is significantly expressed in atherosclerotic plaques of the coronary arteries. Inhibition of PHACTR1 synthesis led to a decrease in the Ox-LDL-induced expression of VCAM-1, ICAM-1, and VE-cadherin; attenuation of p47phox phosphorylation; and attenuation of the p65 and NF-*κ*B activity without affecting I*κ*B*α* and IKK*α*/*β* phosphorylation, all resulting in a decrease of intracellular oxidative stress [145].

Sirtuin 1 (SIRT1) is a protein that plays a role in mitochondrial biogenesis and deacetylation of proteins important for stimulating antioxidant defense. SIRT1 enhances the antioxidant enzyme activity and inhibits free radical-mediated oxidative injury by reducing NADPH oxidase activation, also reducing endothelial cell death caused by oxidative injury [146]. The main mechanism of its action is the inhibition of the LOX-1 expression by modulation of the LOX-1 promoter [147]. The SIRT1 expression level is suppressed, while the acetylated p53 expression levels are increased in monocytes of CAD patients. The mitochondrial function is significantly impaired in monocytes in patients with CAD, and it is thought that SIRT1 may increase the mitochondrial function. Also, a consequence of the decreased expression of SIRT1 is the increased adhesion of monocytes to endothelial cells [148].

We can conclude that oxidative stress plays a key role in the development and pathogenesis of CAD and the emergence of its complications [2]. By modulating very complex and numerous singular pathways and various biomolecules, oxidative stress can be reduced. In the future, there is a possibility and need to investigate more thoroughly all molecules involved in this highly complex biological process, which may open up new therapeutic targets and ultimately reduce the onset and complications of CAD.

## 6. Coronary Microvascular Dysfunction

**Figure 4:**
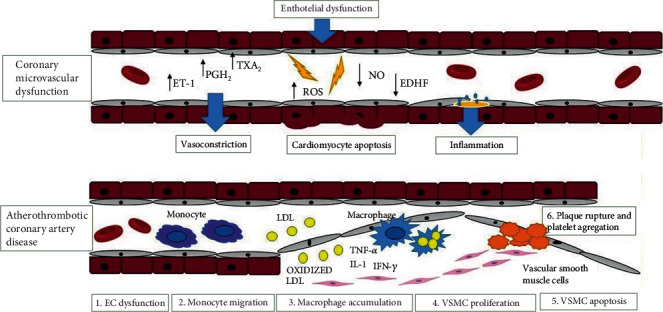
Difference between coronary microvascular dysfunction and atherothrombotic coronary artery disease. ET-1: endothelin -1; PGH2: prostaglandin H2; TXA2: thromboxane A2; ROS: reactive oxygen species; NO: nitric oxide; EDHF: endothelium-derived hyperpolarizing factor; LDL: low-density lipoprotein; TNF-*α*: tumor necrosis factor – *α*; IL-1: interleukin-1; IFN- *γ*:), interferon-gamma; VSMC: *vascular smooth muscle cells;* EC: endothelial cell.

Coronary microvascular dysfunction (CMD) is a disorder that leads to the development of myocardial ischemia, although there is no proven obstruction in coronary arteries on coronary angiography [149] (Figure 4). Risk factors that can trigger oxidative stress in coronary microvascular dysfunction are obesity, dyslipidemia, diabetes, and the metabolic syndrome. Some disorders such as hypertrophic cardiomyopathy, hypertensive heart disease, myocarditis, and vasculitis are examples where myocardial ischemia can develop without the presence of coronary artery obstruction. In addition to these conditions, structural and functional alterations in the coronary microcirculation may be responsible for the occurrence of myocardial ischemia, in up to 20% of patients with acute coronary syndromes (ACS) and up to 50% of patients with chronic coronary syndromes (CCS) [150].

### 6.1. CMD in Nonobstructive ACS

MINOCA is a term that refers to myocardial infarction with nonobstructive coronary arteries [13]. Today, the pathophysiology of MINOCA is not very well understood. Some studies show that MINOCA has two causes: epicardial causes which are represented by coronary plaque disease, coronary dissection, coronary artery spasm and micorvascular causes such as coronary microvascular spasms, Takotsubo syndrome, myocarditis, or coronary embolism [17, 151]. Conditions such as myocarditis and Takotsubo syndrome are considered nonobstructive ACS, but cardiac nonischemic aetiologies [152, 153].

### 6.2. CMD in Nonobstructive CCS

INOCA is a term denoting ischemia with non-obstructive coronary arteries, where endothelial dysfunction is a key mediator in the pathogenesis of CMD [154, 155]. Studies have shown that INOCA is present in approximately one-third of men and two-thirds of women undergoing angiography for suspected ischemic heart disease [155, 156]. Some studies show that factors originating from the blood and endothelium, as well as metabolic and neurohumoral influences, affect the regulation of the coronary microvascular tone. These include the influence of passive mechanical factors (extravascular contraction of contracting myocardium, distension by intravascular pressure) as well as active changes in the smooth muscle tone by myogenic responses (in response to changes in perfusion pressure) [157, 158]. An important role in the development of this disorder is played by the vascular endothelium where if there is dysfunction, inadequate release of NOS would result in coronary artery vasoconstriction [149]. More specifically, reduced endothelial NO synthesis or increased inactivation will result in endothelial dysfunction and vasoconstriction of blood vessels [159–161]. Endothelial dysfunction is also present as an imbalance between the release of vasorelaxant substances, such as prostacyclin (PGI2), endothelium-derived hyperpolarizing factors (EDHF), and vasoconstricting substances, such as endothelin-1, superoxide, hydrogen peroxide, and thromboxanes [162]. Endothelin-1 (ET-1), as a potent vasoconstrictor, plays a significant role in the pathogenesis of coronary microvascular dysfunction by acting through endothelin A receptors located on coronary vascular smooth muscle cells. Also, ET-1 participates in the regulation of vascular tone via endothelin B receptors located on coronary vascular smooth muscle cells and on endothelial cells where it has an effect on NO release and vasodilation [163]. Endothelium-derived NO is produced from *L*-arginine using NO synthase and released to the vascular smooth muscle layer, ultimately causing vasodilation. NO occurs in response to an increase in shear stress [164]. Endothelial NO has an effect on mitochondrial metabolism, reducing the production of ROS and thus inhibiting inflammation. In addition, it inhibits myocyte hypertrophy by activating cGMP-dependent protein kinase (PKG). It also prevents thrombosis and vascular inflammation by inhibiting platelet activation. Therefore, in conditions such as ischemia and metabolic diseases, there is an increased release of ET-1, thromboxane A2, and ROS, which ultimately results in increased cardiomyocyte apoptosis [165].

Corban et al. have combined numerous studies, pointing out that mutations of eNOS and ET-1 genes are crucial for the development of coronary microvascular dysfunction [162]. For example, an eNOS gene missense Glu298Asp variant is associated with reduced NO production and impaired endothelial cell response to physiological stimuli such as shear stress, then the T786 > C mutation in the eNOS gene compromises endothelial NO synthesis [166, 167].

Ford et al. conducted a multimodality investigation on patients with angina, investigating the role of ET-1 and the gene variant (rs9349379-G allele), chromosome 6 (PHACTR1/EDN1)] in the pathogenesis of CMD. Their goal was to investigate whether the G allele associates with noninvasive parameters of myocardial ischaemia. The second goal was to examine vascular mechanisms using isometric tension recordings in small peripheral resistance vessels isolated from patients according to genotype. In conclusion, peripheral small artery reactivity to endothelin-1 and ETA receptor antagonist affinity was conserved in the rs9349379-G allele group. Zibotentan tested at clinically relevant concentrations completely prevented the effect of endothelin-1. This study indicates that ETA receptor antagonism in this group of patients may have therapeutic benefits [168, 169].

Experimental studies conducted to date on large animal models such as swine, given that they show a remarkably similar cardiovascular anatomy as humans, have significantly helped in the understanding of the regulation of coronary microvascular function [170]. Experimental studies on animal models, with an emphasis on metabolic derangements as risk factors—in dogs, swine, rabbits, rats, and mice—today help to understand the pathophysiology of CMD. Metabolic derangements in animals are most commonly caused by a high-fat diet (HFD) and/or diabetes mellitus through an injection of alloxan or streptozotocin. There are also transgenic animal models in which metabolic derangements develop. All these animal models show disturbances in the function and structure of the coronary microvascular bed. Therefore, the application of these animal models will be useful in identifying novel therapeutic targets for the purpose of combating ischemic heart disease [171]. Experimental studies have shown that adipocytes, leptin, interleukin-6 (IL-6), and tumor necrosis factor-*α* (TNF-*α*) are crucial in the development of oxidative stress [172, 173]. In patients with metabolic syndrome, the increased sympathetic activity produces exaggerated alpha-adrenergic coronary vasoconstriction and thus contributes to the development of coronary microvascular dysfunction [174]. Also, in patients with metabolic syndrome and prehypertension, the RAAS system is activated resulting in the formation of angiotensin II—causing vasoconstriction in the coronary circulation [175].

In spite of the conducted research efforts to date, there is still insufficient knowledge about the role of oxidative stress in the pathophysiology of coronary microvascular dysfunction, as a disorder leading to the development of myocardial ischemia despite a normal finding of coronary angiography.

## 7. The Impact of Environmental Factors on CAD

Research to date has shown that environmental factors may play an important role in the development of cardiovascular disease (CVD), but the mechanisms by which environmental factors affect CVD have not been fully explained [176]. Knowing how different environmental factors affect CVD risk would greatly improve the development of therapeutic and preventive strategies to combat CVD. In addition to the previously known fact that genetics, combined with environmental factors, is contributing to the development of CVD, the results of many studies have shown that environmental factors play a more dominant role, as many subjects have prevented CVD by maintaining a healthy lifestyle [177].

The study by Hill et al. investigated the influence of selected genetic and environmental factors on the clinical expression of heterozygous familial hypercholesterolemia. Men were shown to have a higher risk of developing CAD because they had lower high-density lipoprotein (HDL) cholesterol levels and were smokers. In women, CAD has been associated with elevated triglycerides and the presence of hypertension [178].

In order to understand how the environment affects CVD or how that risk is transmitted, we need to understand the complexity of the human environment. According to research, it has been shown that there is a mismatch between ancient human genes and the current human environment, and that the mismatch is the result of a rapid change in the human environment relative to genetic adaptation. First of all, the circadian rhythm is a fundamental feature of the natural environment and has an impact on the levels of neurohormones that regulate cardiovascular function, such as angiotensin II, renin, aldosterone, growth hormone, and atrial natriuretic peptide [179–181]. Therefore, an interesting link is that the frequency of adverse cardiovascular events varies with time. For example, myocardial infarction most commonly occurs between 6 a.m. and 12 p.m. and is more likely to occur early in the morning than at night [182, 183]. Also, a disturbed circadian rhythm increases the risk of diabetes mellitus, obesity, and hypertension [184–186].

The change of seasons has an impact on the development of CVD which is shown by research which found that in the northern and southern hemispheres, and the levels of blood pressure, HDL, LDL, and glucose are slightly higher in winter than in summer. More patients on statin therapy reach the target LDL level in summer than in winter [187–189]. Likewise, exposure to cold ambient temperature increases vascular resistance and blood pressure and can induce coronary vasospasm and lead to the development of myocardial infarction [190]. Also, heat waves, especially in the elderly who cannot adapt quickly to changes in temperature, can promote the development of CVD [191]. That high levels of sunlight early in life can delay CVD by 0.6 to 2.1 years has been shown by some studies [192, 193]. Vitamin D deficiency is associated with an increased risk of adverse cardiovascular events such as myocardial infarction, stroke, heart failure, and sudden cardiac death [194, 195].

Studies have shown that short-term irradiation of the whole body of healthy people with UVA has the effect of lowering blood pressure, on the principle of releasing NO, and increasing the level of S-nitrosoglutation, which reduces blood pressure [196–200]. Also, differences in solar exposure to UV radiation and synthesis of vitamin D increase at high altitudes [201]. Studies show that the proximity of vegetation is associated with lower levels of stress, diabetes mellitus, and CVD [202, 203]. Children who live in greener areas have lower levels of asthma, blood pressure, and insulin resistance [204, 205]. Socioeconomic conditions have an impact on CVD as evidenced by higher data on the incidence of the disease among the poor population. Which is also related to the supply of food and the availability of health care [206]. Exposure to synthetic chemicals and environmental pollutants can have an impact on the health of the population and is ubiquitous and unavoidable today [207]. There is evidence to suggest that chronic and persistent exposure to air pollution increases the progression of atherosclerotic lesions and has adverse effects on blood pressure regulation, peripheral thrombosis, endothelial function, and insulin sensitivity [201, 208–210]. Some studies have shown that constant exposure to noise induces stress and has an impact on cognitive function, autonomic homeostasis, and sleep, and that it increases the risk of CVD [211]. In animal models, chronic exposure to continuous noise (80-100 dB) has been shown to increase the heart rate and mean systemic arterial blood pressure, functional changes associated with increased plasma corticosterone, adrenaline, and endothelin-1 [212].

Smoking, as one of the environmental factors, has a great influence on the development of CVD. Data show that smoking reduces regional left ventricular function even in asymptomatic individuals and significantly (45% –80%) increases the risk of heart failure [213]. The reasons for the high vulnerability of cardiovascular tissue remain unclear, but may relate to poor xenobiotic metabolism in these tissues and their direct exposure to blood-borne toxins. Although the mechanisms by which smoking increases the risk of CVD are not fully known, they appear to affect CVD independently of other factors [214]. A meta-analysis of 54 different studies suggests that smoking increases LDL-C and decreases HDL, but lipid changes account for <10% of the excessive risk of CVD in smokers [215]. Similarly, although acute smoking affects blood pressure, smokers tend to maintain lower blood pressure. Smoking leads to coronary occlusion causes endothelial dysfunction and platelet adhesion to subintimal layers, thereby increasing lipid infiltration and platelet-derived growth factor- (PDGF-) mediated proliferation of smooth muscle cells [216].

Studies have shown that people with homocystinuria, which is one of the inherited recessive disorders in methionine metabolism, have a tendency to develop cardiovascular disease. Such persons have high levels of homocysteine in the circulation and urine, which has an impact on the development of atherosclerosis and in the coagulation system [217–219].

Also, patients with hyperuricemia have a tendency to develop CAD because serum uric acid levels are positively associated with arterial intima-media thickness, which is a precursor to atherosclerosis [220, 221]. In conclusion, we can greatly contribute to the prevention and severity of CVD by influencing environmental factors.

## 8. Pharmacological Therapeutic Possibilities

The therapeutic approach in patients with or without evidence of coronary atherosclerosis involves, first and foremost, lifestyle changes and the management of risk factors, including an effort to influence environmental factors. Beta-blockers are a class of medications that are used to protect the heart from a myocardial infarction because they may reduce myocardial oxygen consumption [222]. Potential therapeutic strategies are focused on the NO-cGMP (nitric oxide-cyclic guanosine monophosphate) pathway. Given that the NO-cGMP pathway has been implicated in the pathophysiology of heart failure, it is a promising target for therapy; although unfortunately, clinical data are not yet fully conclusive [222]. A beta-blocker such as nebivolol exerts its effect through beta-adrenoreceptors located on endothelial cells. In this way, it stimulates eNOS, which ultimately results in NO release and vasodilation. Data on the effect of nebivolol have been supported by studies such as the SENIORS (the Study of the Effects of Nebivolol Intervention on Outcomes and Rehospitalization in Seniors with Heart Failure) study conducted in elderly patients with heart failure [223–225].

Mihai et al. investigated the effect of vericiguat, a soluble guanylate cyclase (sGC) stimulator, on N-terminal prohormone of brain natriuretic peptide (NT-proBNP) levels in patients with chronic heart failure and reduced ejection fraction. The study concluded that among 351 patients with heart failure (HF) and reduced ejection fraction, compared with placebo, vericiguat did not have a statistically significant effect on NT-proBNP levels at 12 weeks. Therefore, the researchers suggested additional clinical trials of vericiguat based on the dose-response relationship to determine the potential role of this drug, and that phase III outcome trial is still ongoing [222]. Natriuretic peptides (NPs) via the natriuretic peptide receptor-A are known to increase intracellular cyclic guanosine monophosphate (cGMP) levels [226]. A drug such as sacubitril/valsartan that simultaneously inhibits neprilysin (neutral endopeptidase) via LBQ657 and the angiotensin II receptor has its effect in chronic heart failure with a reduced ejection fraction. The benefits of this drug are attributed to the increase in the amount of peptides that neprilysin breaks down, such as NPs, by LBQ657 and the simultaneous inhibition of the effects of angiotensin II by valsartan. NPs exert their effects by activating membrane-bound receptors paired with guanylate cyclase, which results in an increase in the second messenger cGMP and ultimately leads to vasodilation, natriuresis, diuresis, and decreased sympathetic activity. These insights are supported by the PARADIGM-HF administration trial [227]. Also, research such as PARAMOUNT, designed as a randomized, parallel-group, double-blind study in a phase II clinical trial of sacubitril/valsartan in the clinical syndrome of HF with preserved ejection fraction (HFpEF), suggested benefits in HFpEF at least in terms of NT-proBNP reduction [228].

Medication groups such as angiotensin-converting enzyme (ACE) inhibitors and statins are used in patients who have evidence of endothelial dysfunction and evidence of atherosclerosis. ACE inhibitors exert vasoprotective effects by inhibiting the renin-angiotensin axis. Statins, in addition to reducing cholesterol levels, also have an inhibitory effect on vascular inflammation, they upregulate eNOS, and enhance vascular NO bioavailability [229].

Studies to date have shown that antioxidants such as flavonoids and vitamins reduce the risk of stroke [230, 231]. Since ROS are known to occur during ischemia, reperfusion, and bleeding in the brain, several antioxidants of different chemical structures have been investigated as neuroprotective therapeutic agents for brain injuries. An example of this is the use of Vaccinium berries that have high antioxidant activity and that have been used in an animal model. They showed their neuroprotective effect due to the high total content of polyphenols [232–234]. It would be interesting to consider such antioxidants in ischemic heart disease, although conclusive evidence is lacking for now.

Resveratrol (chemical name: 3,5,4′-trihydroxy-trans-stilbene) is another polyphenol abundantly found in the skin and seeds of grapes [235, 236].

NXY-059 (chemical name: *α*(2,4-disulfophenyl)-N-tertbutylnitrone) is a novel nitrone free radical trapping (antioxidant) agent. This compound is a stable form of NO, capable of inhibiting the reaction of O2 - and NO to produce ONOO-. This chemical agent might thus be able to neutralize ROS [237, 238].

Therapeutic options for CMD are limited. Some studies show that inhibition of Rho-kinase might constitute one of the treatment options in patients with CMD and vasospastic angina, but this has not yet been proved [239]. Some studies show that targeting of perivascular adipose tissue to stimulate the production of vasoactive factors such as hydrogen sulphide [240] and adiponectin could be of benefit [241].

Studies show that the use of platelet inhibitors such as aspirin may have an effect on treatment in CAD but they have not been sufficiently implicated in the treatment of CMD [241]. Studied of Zhang et al. showed aspirin provides a new potential strategy for regulating cardiac microcirculation, preventing heat stress- (HS-) induced heart failure. In this study, they used a heat stress model of rat cardiac microvascular endothelial cell cultures in vitro and investigated the cell injuries and molecular resistance mechanisms of cardiac microvascular endothelial cells (CMVECs) caused by heat stress. In conclusion, aspirin treatment of CMVECs induced a significant expression of heat shock proteins (Hsp90), which promoted both Akt and M2 isoform of pyruvate kinase (PKM2) signals, which are beneficial for relieving HS damage and for maintaining the function of CMVECs [242].

Clinical research on the use of ticagrelor for microcirculation protection is still ongoing [243]. Nitrates are effective in inducing vasodilatation, and they relieve angina symptoms, but not in patients with nonobstructive CAD [244]. *L*-arginine is as precursor of NO, with attempted use in subjects with nonobstructive CAD [245, 246], but its use is controversial. Zibotentan and atrasentan are ETA receptor antagonists, and there are studies that have suggested them to be a potential therapeutic option in patients with microvascular dysfunction [247, 248].

Drugs or substances that modify TLR4 signaling can be very useful in treating the atherosclerotic process in the coronary arteries [249]. Some already known cardiovascular drugs may have pleiotropic anti-inflammatory and antiatherosclerotic effects achieved through TLR4 (). The well-known statin atorvastatin [249] and angiotensin-converting enzyme (ACE) inhibitors fosinopril [250] showed their antiatherosclerotic properties because they reduced the expression of the TLR4 protein in atherosclerotic lesions. Furthermore, combination treatment with atorvastatin and telmisartan (angiotensin II receptor blocker) or atorvastatin and enalapril (ACE inhibitors) in human PBMCs (peripheral blood mononuclear cells) resulted in decreased TLR4 receptor expression in patients with CAD [251]. Some studies have shown that thiazolidinediones (TZDs), such as rosiglitanose and pioglitazone, can exert their antiatherogenic effect by inhibiting the TLR4 singular pathways [252–254]. Carvedilol, a third-generation beta-blocker, decreased the TLR4 expression in AIM-induced rats [255]. Paclitaxel, an anticancer drug, has also been shown to inhibit TLR4 signaling [256]. The anesthetic propofol and ketamine have the ability to reduce ROS production and suppress the NF-*κ*B expression and reduce IL-6 [256]. The exact mechanisms of action of these already known cardiological drugs remain to be explored in the future. Of course, there are a number of newly discovered potential TLR4 antagonists (eritoran, cyanobacterial product (CyP), EM-163, epigallocatechin-3-gallate, 6-shogaol, cinnamon extract, N-acetylcysteine, melatonin, molecular hydrogen, monoclonal antibody anti-hTLR4-IgG) which could be useful in preventing atherosclerosis in patients with CAD [257].

Also, epigenetic regulation (DNA methylation and histone acetylation) could become the most promising therapeutic target for the treatment of TLR4-mediated inflammatory disorders [258].

Tsai et al. conducted research in rat and in vitro models examining the role of IL-20 in the infarcted heart following ischemia/reperfusion injury, with the aim of discovering new therapeutic options in the treatment of ischemic heart disease. This study revealed that IL-20 and its receptors, IL-20R1 and IL-20R2, were increased in H2C2 cardiomyoblast cells and ventricular tissues subjected to prior hypoxia/reoxygenation (H/R) stimulation. The obtained results suggest that IL-20 causes an increase in Ca^2+^ and activation of the PKC/NADPH oxidase pathway, leading to an increase in oxidase stress and a decrease in AKT regulation. Also, IL-20 can mediate H/R-induced apoptosis via PKC/NADPH oxidase/AKT signaling. Therefore, regulation of IL-20 may contribute to cardiomyocyte apoptosis, and this might be helpful in future considerations of new therapeutic targets in the treatment of ischemic heart disease [259]. In their work, Samakova et al. combined insights into the phosphatidylinositol-3-kinase- (phosphoinositide-3-kinase-) protein kinase B (serine-threonine protein kinase) (PI3k/Akt) pathway and the association with oxidative stress, angiogenesis, and mesenchymal stem cell survival in pathophysiologic conditions in ischemia [260].

Cell therapy has long been known to be one of the options for treating ischemic heart disease when, in 1974, Friedenstein and his associates first isolated and characterized the use of mesenchymal stem cells (MSC) [261, 262]. Since then, numerous studies have been conducted to improve the use of mesenchymal stem cells in regenerative therapy. Also, the influences of biologically active molecules such as cytokines, growth factors, and chemokines were found to be important for any attempts at successful cell therapies. In addition, the PI3K/Akt pathway was determined to be one of the mechanisms of intracellular signaling that plays a role in regulating cell proliferation, differentiation, apoptosis, and migration. Therefore, the aforementioned contributors emphasized that preconditioning of MSCs is an important process for the improvement of the efficiency of signaling mechanisms [260].

Some studies show that fisetin protects against cardiac cell death through reduction of ROS production and caspase activity. In vitro studies of mammalian cardiac cell models have shown that fisetin increases the vitality of rat cardiomyocytes after hypoxia or starvation or reoxygenation. It also reduces ROS formation, activates caspases, protects from DNA damage, and ultimately inhibits apoptosis. Fisetin is a very promising drug for protection against ischemic damage after myocardial infarction and for counteracting ischemia reperfusion injury because it can, in addition, activate genes involved in cell proliferation [263].

Experimental studies in rat models have shown that cocoa flavonoids reduce inflammation, oxidative stress, and myocardial apoptosis after acute coronary ischemia-reperfusion. In these studies, cocoa extract treatment reversed membrane peroxidation and nitro-oxidative stress as well as lead to reduction of inflammatory marker levels such as IL-6 and NF-*κ*B [264]. Verma et al. conducted an experimental study in rats that showed that morin, a bioflavonoid, has antioxidant and anti-inflammatory effects, and that it prevents apoptosis. It exerts its effects by regulating RISK/SAPK pathways. Extracellular regulated kinase (ERK), protein kinase A (Akt), and eNOS are involved in the RISK pathways. The p38 proteins and c-Jun N-terminal kinase (JNK) are involved in the SAPK pathway [265]. Syeda et al. in their study in mice investigated the cardioprotective potential of anthocyanidin against myocardial ischemia injury. In in vivo conditions, the left anterior descending coronary artery was ligated to induce myocardial ischemia in mice, whereas in in vitro conditions, neonatal mice cardiomyocytes were treated with H_2_O_2_ to induce oxidative stress. It was concluded that, in vivo and in vitro, anthocyanidin can induce a state of myocardial resistance against ischemic insult. Inhibition of the ROS/p-JNK/Bcl-2 pathway is the underlying mechanism of action of anthocyanidin [266].  Table 1 summarizes the discussed pharmacological therapeutic possibilities.

## 9. Biomarkers of Oxidative Stress in Ischemic Heart Disease

Many oxidative stress-related biomarkers have been recently proposed, reflecting different and independent pathways, including oxidative and antioxidant ones [267]. Some reliable and simple tests have been presented to estimate oxidative stress in vivo, and also a calculation of a global oxidative stress index (OSI) is described, which represents the ratio of total oxidant status to total antioxidant status [268], and it showed higher values in patients with CAD [269]. Some of the oxidative stress-related biomarkers seem promising for future clinical use in understanding the pathogenesis and predicting clinical outcomes of ischemic heart disease. Although there are a lot of common biological features between ACS and stable CAD, there are also many differences resulting in variation of levels of biomarkers included in different oxidative stress-related pathways [270].

Measurement of reactive oxygen metabolites (ROM) based on the conversion of hydroperoxides to alkoxyl and peroxyl radicals under acidic conditions in combination with estimation of total antioxidant capacity (OXY) can quantify oxidative stress levels [271, 272]. Previous studies evaluated levels of ROM and OXY in patients with cardiovascular disease in comparison with the general population and evaluated their prediction value in adverse CV events [268, 271, 273]. Lubrano et al. examined ROM and OXY levels during acute myocardial infarction (AMI) which showed a progressive increase and then decrease suggesting significant rise of oxidative stress level during AMI [270]. The level of ROM values was higher in stable CAD in comparison with ACS patients, indicating that this parameter reflects the chronic oxidative stress status [270]. OXY was progressively reduced in stable CAD and more in ACS compared with the control group, showing severe acute harm to the antioxidant system in ischemic disease, especially during myocardial reperfusion injury [270]. This fact is further enhanced by findings that different vitamins and antioxidant enzymes were also reduced during acute myocardial infarction [270, 274].

Low levels of NO are related to endothelial dysfunction and many CV events, but its direct quantification is difficult so it can be estimated by measurements of its stable metabolites—nitrite/nitrate (NOX) [275]. NOX are end-products of NO metabolism and a reliable index of NO production. In previous studies, there are controversial results regarding NOX levels in CV disease and CV risk. Some of them revealed higher levels of NOX in a group with CAD and AMI, which can be explained by the fact that increase in systemic NOX can be a consequence of activation of inducible NO synthase as a result of vascular injury, without restoration of endothelial NO release [276, 277]. Other studies showed reduced levels of NOX during acute myocardial infarction, pointing to deteriorated NO levels during an acute ischemic event. Further researches are needed to understand meaning of different levels of NOX [270].

Several studies suggested that Ox-LDL may play an important role in the pathogenesis of atherosclerosis, plaque rupture, and onset of ACS [278, 279]. Uptake of Ox-LDL by macrophages and activated smooth muscle cells probably transforms these cells into foam cells which are found in the atherosclerotic intima. Endothelial uptake of Ox-LDL depends on receptors expressed on the cell surface. LOX-1 is a major receptor for Ox-LDL, and its expression is induced by oxidative stress, hemodynamic stimuli, and inflammatory cytokines, and it is related to development of atherosclerosis and plaque instability. It is highly expressed in luminal endothelial cells in the early stage of atherogenesis as well as in intimal neovascular endothelial cells of advanced plaques and released during plaque rupture, promising to be a good marker of plaque instability [280]. Soluble LOX-1 (sLOX-1) is proposed as a potential marker for identification of ACS in the early stage [281, 282] with the peak time being even earlier than troponin T [281]. Some studies suggest that levels of sLOX-1 might begin to rise before onset of ACS but that could be a subject of further research [282].

In addition to these biomarkers, there is a large cohort study that found an association of urinary oxidized guanine/guanosine (OxGua) and 8-isoprostane levels with CVD mortality prediction and with myocardial infarction incidence in obese subjects [283]. Other studies also showed that elevated plasmatic levels of 8-isoprostane are associated with acute myocardial infarction and also with the severity and extent of CAD [284, 285].

## 10. Epigenetic (Dys)regulation MicroRNA in CAD

**Figure 5:**
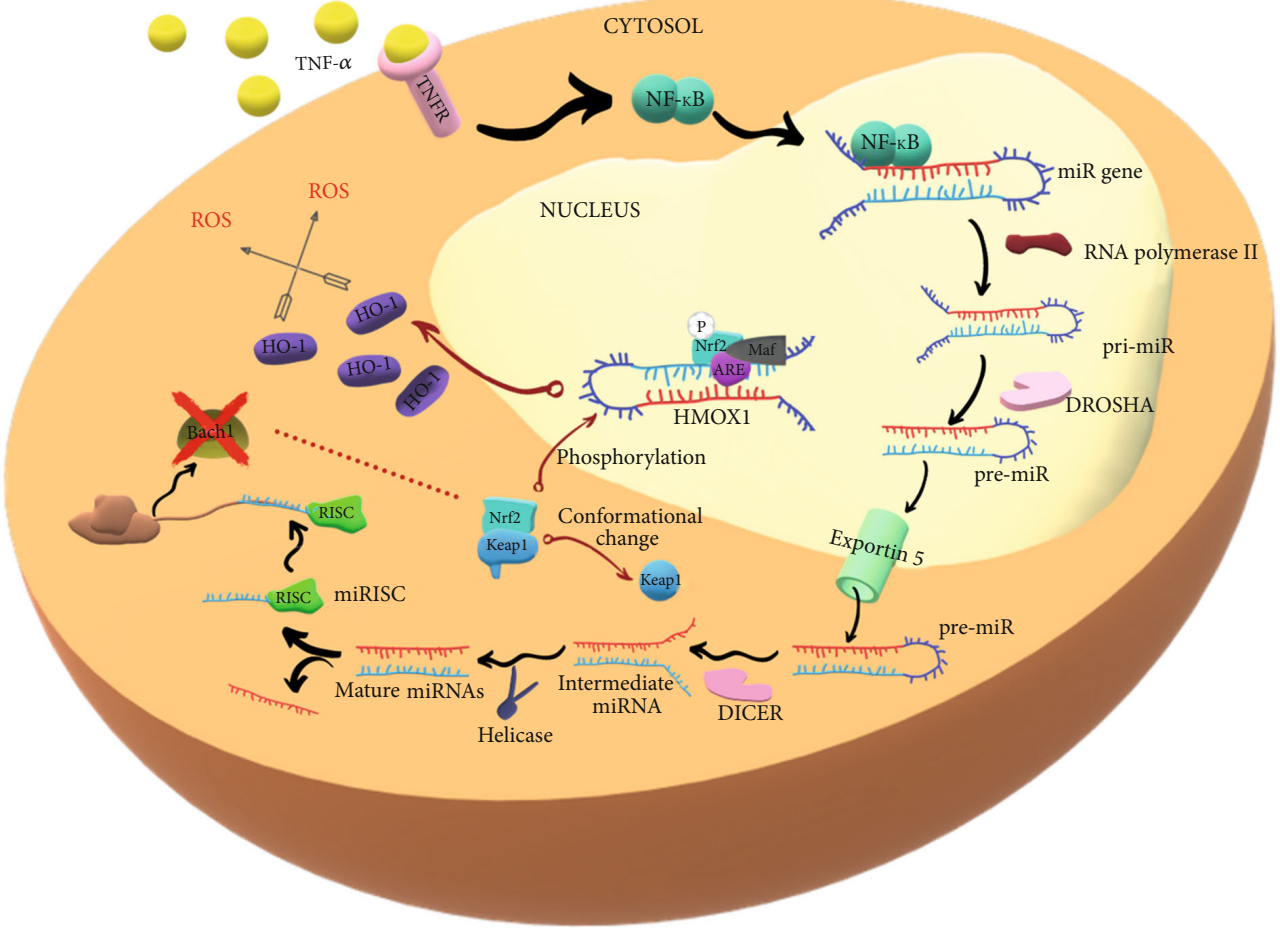
Biogenesis of miR-155. ARE: antioxidant responsive element; Bach 1: BTB domain and CNC homolog 1; DICER: ribonuclease DICER; DROSHA: ribonuclease DROSHA; HO-1: heme oxygenase-1; KEAP1: Kelch-like ECH-associated protein 1; MAF: musculoaponeurotic fibrosarcoma; miR: microRNA; NF-*κ*B: nuclear factor kappa-light-chain-enhancer of activated B cells; NRF2: nuclear factor erythroid 2-related factor 2; RISC: RNA-induced silencing complex; ROS: reactive oxygen species; TNF-*α*: tumor necrosis factor-*α*; TNFR: tumor necrosis factor receptor.

Small noncoding RNAs (microRNAs or miRs) of 19–25 nucleotides (nt) regulate the expression of more than 30% of human genes at the posttranscriptional level [286]. They are involved in intracellular and intercellular signaling and circulate in the blood in stable forms due to packaging into apoptotic bodies, microvesicles (MV), exosomes, and lipoproteins (Lp) [287]. Each miR from RNA is transcribed by RNA polymerase II and, less frequently, by RNA polymerase III [288, 289]. First, Pri-miRNA is formed, containing a canonical hairpin structure, a 50 cap, and a 30 poly-A tail, which is processed in the nucleus by Drosha-DGCR8 (Di George Syndrome Critical Region Gene 8), then pre-miRNA is formed to a hairpin form which is exported to the cytoplasm via Exportin-5 and then further truncated by the RNase III enzyme complex Dicer/TRBP (TAR RNA-binding protein), yielding a mature duplex of miRNA (miRNA-5p and miRNA3p) [290]. Mature miRs bind at a specific site of the messenger RNA (mRNA) in the Argonaute protein multiprotein complex, known as RNA-induced attenuation complex (RISC), providing sequence-specific attenuation by degrading messenger RNA (mRNA) and/or inhibiting its translation [286]. Each miR can target several mRNAs that act in several important cellular functions such as differentiation, proliferation, and apoptosis in the cardiovascular system [291]. MicroRNAs that can regulate cellular homeostasis of oxidative stress by modulating the expression of antioxidant genes and the expression of enzymes that generate ROS are called redox sensitive micorRNAs or redoximiR [292]. RedoximiR achieves its oxidative or antioxidant effects by directly regulating the posttranscriptional level of the redox-sensitive nuclear factor Nrf2. Of course, there are also miRs that achieve their effects independently of Nrf2 [293]. Nrf is considered a major regulator of cell survival in oxidative stress because it controls the basal and induced expression of a number of important antioxidant genes via a *cis*-acting element, designated the antioxidant-response element (ARE), in the promoter of target genes [293]. Some of these genes are the genes for heme oxygenase-1 (HO-1), gamma-glutamylcysteine synthetase, thioredoxin reductase, glutathione-S-transferase, and NAD (P) H: quinone oxidoreductase [293]. Protein kinase C (PKC), mitogen-activated protein kinase (MAPK), and phosphotidylinositol 3-kinase (PI3K) are involved in the regulation of Nrf2/ARE signaling [293]. Numerous microRNAs participate in the regulation of Nrf2 via certain coregulatory proteins such as Kelch-like ECH-associated protein 1 (Keap1), BTB and CNC homolog 1 (Bach1), Parkinson's protein 7 (PARK7/DJ-1), and small masculoaponeurotic fibrosarcoma (Maf) proteins. Most miRs act by the downregulation mechanism Nrf2 (miR-153, miR-27a, miR-142-5p, miR144 miR-28, and miR-34a), while some act by the upregulation mechanism Nrf2 (miR 200-a, miR-136-3p, miR-128). Also, miRs regulate the expression of key enzymes that generate ROS which can lead to modification of the biogenesis of miRs. Cellular oxidative stress can alter miR biogenesis during processing in the nucleus and cytoplasm, altering its stability, functionality, and binding affinity for target promoter sites [292] (Figure 5).

The primary pathological process that causes CAD is atherosclerosis triggered by oxidative stress [291]. miR is involved in almost all steps of atherosclerosis and CAD, such as endothelial damage and endothelial dysfunction, oxidative enzyme expression, inflammatory molecule expression, monocyte invasion and activation, LDL oxidation, platelet function, vascular smooth muscle response, and angiogenesis [294]. The miRs involved in oxidative stress and associated with CAD are miR-155, miR34a, and miR-136-3p.

miR-155 was expressed in mononuclear and endothelial cells [295]. Ox-LDL can induce the expression of miR-155 [295]. miR-155 may inhibit the inflammatory response thereby reducing enhanced lipid oxidation in macrophages during oxidative stress [295]. Inhibition of endogenous miR-155 in THP-1 macrophages resulted in increased lipid uptake and release of several cytokines, including interleukin (IL)-6, -8, and tumor necrosis factor-*α* (TNF-*α*) [296]. The overexpression of miR-155 may induce apoptosis of Ox-LDL attacked macrophages [295]. Levels of miR-155 were decreased in plasma and peripheral blood mononuclear cells (PBMCs) in patients with CAD. The study showed that miR-155 levels in peripheral blood mononuclear cells or plasma were inversely correlated with the severity of stenotic lesions in the coronary arteries [297]. miR-155 acts on the Nrf2 pathway whose activation of Nrf2 can reduce the degree of oxidative stress in mitochondria and can reduce oxidative stress and the inflammatory response of vascular endothelial cells [298]. Bach1 in association with the masculoaponeurotic fibrosarcoma (Maf) protein dominantly hides ARE sequences from Nrf2 binding of transcription factor [299, 300]. Oxidative stress and inflammation-induced tumor necrosis factor-*α* (TNF-*α*) activate TNFR (tumor necrosis factor receptor) [301]. NF-*κ*B is in an inactive state bound to the inhibitor protein I*κ*B, and activation of TNFR leads to activation of I*κ*B kinases (IKKs) which by phosphorylation dislocates I*κ*B with NF-*κ*B [302]. Phosphorylated IB is degraded by the 26s proteasome [303]. Nuclear translocation of NF-*κ*B stimulates the expression of miR-155 which inhibits the production of Bach1 protein, allowing the binding of the Nrf2 transcription factor to the ARE sequence [299]. Under normal physiological conditions, Nrf2 is bound to Kelch-like ECH-associated protein-1 (Keap1) within the cytoplasm [304].When the Bach1 expression is reduced and cells are attacked by oxidative stress, phosphorylation of Nrf2 via (MAPK), protein kinase C (PKC) and (phosphoinositide 3-kinase (PI3K) and its transport into the nucleus and binding to the ARE sequence together with Maf protein occurs [295]. Consequently, there is an increased synthesis of HO-1 (heme oxygenase-1) [299]. HO-1 is a microsomal enzyme induced in oxidative stress that metabolizes heme to biliverdin, carbon monoxide (CO), and iron, and CO has antiapoptotic and anti-inflammatory properties and may act as a vasodilator in atherogenesis when NO bioavailability is reduced due to ROS inactivation [305]. Numerous studies have shown a cardioprotective effect of HO-1 [306–308]. Thus, it is clear that suppression of the Bach1 protein expression alters cellular redox signaling and enhances the expression of antioxidant enzymes induced by Nrf2 [309].

miR-34a induced by oxidative stress via PI3K signaling in EPCs obtained from patients with CAD reduces the expression of the enzymes SIRT1 and SIRT6 involved in histone deacetylation and DNA repair [310, 311]. Silencing the entire miR-34 family may protect the heart from pathological myocardial remodeling [312]. miR-34a induces postacute MI, and inhibition of miR-34a improves recovery of cardiac contractile function after acute MI [313].

miR-136-3p can reduce oxidative stress and inflammatory response and consequent pathological damage to myocardial tissue by inhibiting the expression of the target EIF5A2 gene thereby blocking the Rho A/ROCK signaling pathway in the CAD rat myocardial tissue and models of cardiac microvascular endothelial cell (CMEC) injury [314].

Numerous studies have shown that miR can serve as an important biomarker for early detection of CAD, differentiation of patients with or without CAD, as well as patients with stable CAD or unstable CAD, and assessment of disease severity, as prognostic indicators and indicator of restenosis after stenting (in-stent restenosis, ISR) [291]. miRNAs are not specific because each miRNA can be elevated or decreased in different disease conditions, so this is a big challenge for researchers [291].

Increased levels of miR-31, miR-720, miR-181, and miR-208a may have potential roles for early CAD detection [315–317]. miR-208a is a highly selective cardiac RNA that is overexpressed 3 hours after myocardial infarction (MI) and correlates with increased cardiac troponin (cTn) I levels [318]. MiR-208a has been shown to have superiority in early diagnosis of MI over cTn [318]. Devaux et al. argue that miR-499-5p which is myosin gene-regulated has higher diagnostic accuracy in correlation with cTN than miR-208a [319].

To distinguish CAD from non-CAD patients, numerous miRNAs were detected that were significantly increased in patients with CAD (miR-149, miR-765, miR-424, miR-133a miR-206, miR-574-5p, miR-135a, miRNA-24, miRNA-33, miRNA-103a, miRNA-122). On the other hand, levels of miRNA-23a, miR-19a, miR-484, miR155, miR-222, miR-145, miR-29a, miR-378, miR-342, miR-181d, miR-150, and miR-30e-5p were found to be reduced in the blood of patients with CAD compared to healthy controls [320]. Faccin et al. state that the combination of three miRNAs (miRNA-155, -145, and flight-7c) has better classification power than just one miRNA [321]. Two studies have shown that increased plasma levels of miRNA-133a, miR-126, and miR-1 are useful for the diagnosis of unstable CAD [322, 323]. MiR-145 is significantly elevated in unstable angina compared to stable angina, but so far, no miR or cascade of miR has been detected in the blood of patients by which we will distinguish these two types of angina [287]. Another study showed that miR-134, miR-198, and miR-370 were increased in unstable versus stable angina pectoris [324]. Li suggested that six microRNAs (miR-1/134/186/208a and 208b/233/499-5p) have increased sensitivity and specificity in MI detection, although miR208 and miR499 were significantly higher in patients with pecotris angina compared to IM [325]. Ward et al. demonstrated that myocardial infarction miRNA-25-3p, miRNA-221-3p, and miRNA-374b-5p are highly present in the blood of patients with STEMI and miRNA 221-3p and 483-5 in patient with NSTEMI [326].

To assess the severity of coronary artery disease, miR-133a was presented as a potential biomarker showing the presence of coronary artery stenosis and is a better indicator of assessing the severity of CAD compared to cTn1 [327]. Other miRNA-208a, miRNA-155, and miRNA-223 strongly correlated with the CAD severity assessment [291]. Levels of miRNA-92a lipoprotein-2 (HDL-2) HDL-2 miRNA-92a, and HDL-3 miRNA-486 could be signals of severe CAD and threatened myocardial infarction [328]. Oxidative stress-induced microRNA-92a (miR-92a) leads to endothelial dysfunction caused by activation of sirtuin 1, Krüppel-like factor 2, and Krüppel-like factor 4, leading to NOD-like receptor family pyrin domain-containing 3 inflammasome activation and endothelial nitric oxide synthase inhibition [329]. The expression of miRNA-21 in the macrophages of uncalcified coronary artery lesions was significantly higher than in calcified lesions [330]. miR-100 can be released into the coronary circulation from sensitive coronary plaques and can therefore be useful as a biomarker of plaque vulnerability [331].

Some vascular miRs may have a prognostic potential for coronary artery disease. The increased expression of miRNA-126 and miRNA-199a in circulating microvesicles is associated with a lower cardiovascular mortality rate [332]. Also, elevated levels of miRNA-197, miRNA-223, miRNA-133a, and miRNA-208b were significantly associated with higher mortality rates in patients with CAD [333, 334]. In obese patients, miR-181a levels within polymorphonuclear cells are increased, which is associated with an increased risk of developing CAD [316].

As new noninvasive potential biomarkers for assessing the occurrence of ISR, levels of circulating miRNA-143, miRNA-145, and miRNA-181b and increased levels of miRNA-185 and miRNA-155 were reduced compared to non-ISR [335, 336].

During the development and progression of atherosclerotic plaques, miR-92a, miR-100, miR-126, miR-127, and miR-145 are mostly released as a result of vascular damage. The miRNAs released from myeloid cells involved in the formation of atherosclerotic lesions are miR-155 and miR-223. During myocardial injury in patients with CAD, miR-133a, miR-208a, and miR-499 are mostly released into the coronary circulation [337].

RedoximiR is an important regulator of the cellular redox status and new valuable biomarkers that constitute a key step in the pathogenesis of CAD. Due to their cell-type specificity, abundance, and stability in most solid and liquid clinical specimens, they provide the opportunity for further study to expand our understanding of CAD pathogenesis and open up new innovative diagnostic and therapeutic approaches. For now, the following therapeutic strategies are being studied: inhibition of premicroRNA export from the nucleus, inhibition of premicroRNA transcription into mature microRNAs, or competitive inhibition via complementary binding to specific microRNAs [287]. Whether we can block or prevent the progression of atherosclerosis and CAD development in the future remains to be patiently awaited.

## 11. Final Remarks

This review article discussed evidence associating oxidative stress with the pathogenesis and occurrence of ischemic heart disease. Since oxidative stress is an important group of processes in a number of disorders connected with vascular structure and function, it is not surprising that there are many indications that some of the factors implicated in oxidative stress play roles in vascular disease mechanisms. From this review, however, it is likewise clear that the interactions of the many factors of oxidative stress that contribute to vascular disease mechanisms in ischemic heart disease are very complex and not yet clearly nor completely understood. At the same time, it would be desirable and interesting to therapeutically target oxidative stress, in hopes of developing better therapeutic strategies for ischemic heart disease, which after many years of various treatment approaches and strategy changes is still not managed optimally and with satisfactory results in a large number of affected patients. The main prerequisite for the development of such therapeutic strategies targeting oxidative stress is, however, a much better understanding of all the specific roles of ROS in specific pathophysiological mechanisms, as well as the interactions of ROS with other signaling systems. Only then can a targeted therapeutic approach be successful, effective, and with a limited spectrum of adverse effects. To achieve such an understanding of the roles of oxidative stress in ischemic heart disease, more research in this area is warranted.

## Figures and Tables

**Table 1:** Discussed pharmacological therapeutic possibilities in ischemic heart disease.

Reference	Study characteristic	Therapeutic options	Primary endpoint
Zhang X et al. [242]	Experimental study on animal models such as rat	Aspirin (platelet inhibitors)	Enhances the protection of Hsp90 from heat-stressed injury in cardiac microvascular endothelial cells through PI3K-Akt and PKM2 pathways
Rodius et al. [263]	In vitro studies of mammalian cardiac cell models	Fisetin (plant polyphenol from the flavonoid group)	Reduction of ROS production, protects from DNA damage
Verma et al. [265]	Experimental study on male albino Wistar rats	Morin (bioflavonoid)	Regulation of RISK/SAPK pathways
Syeda et al. [266]	Experimental study on mice	Anthocyanidins (plant pigments)	Inhibition of ROS/p-JNK/Bcl-2 pathway
Flather et al. Ambrosio et al. [223, 224]	Randomized trial in elderly patients with heart failure	Nebivolol (beta-1-selective blocker), beta(3)-adrenoreceptor agonistic effect	Stimulates eNOS, NO release, vasodilatation
Mihai et al. [222]	Randomized trial in patients with heart failure and reduced ejection fraction	Vericiguat (a soluble guanylate cyclase stimulator)	Changes in the NT-proBNP level have not been achieved, but the phase III trial is ongoing
McMurray et al. [226]	Randomized, double-blind trial in patients with heart failure and reduced ejection fraction	Sacubitril/valsartan (NP degradation inhibitor/angiotensin II receptor inhibitor) vs. enalapril	Increase cGMP, vasodilatation
Solomon et al. [227]	Randomized, double-blind study in a phase II trials, in patients with heart failure and reduced ejection fraction	Sacubitril/valsartan (NP degradation inhibitor/angiotensin II receptor inhibitor) vs. valsartan	Changes in NT-proBNP
Carmine et al. [228]	Randomized, prospective, single-blind, placebo-controlled fashion in patients who have chest pain and angiographically normal epicardial vessels	Ramipril (ACE inhibitor) and atorvastatin (statins)	Reduced SOD activity, low superoxide anion level
Amir et al. [245]	Randomized study, double- blind in patients with patients without significant CAD on coronary angiography	*L*-arginine (substrate for NO synthase)	Improve endothelial function, increase NO and NO inhibits the production of endothelin via cGMP pathway
Martin et al. [247]	Single-center, double-blind, randomized controlled trial in patients with CMD	Atrasentan (ETA receptor antagonist)	Supports the role of the endogenous endothelin system

eNOS: endothelial nitric oxide synthase; NO: nitric oxide; NT-proBNP: N-terminal prohormone of brain natriuretic peptide; cGMP: cyclic guanosine monophosphate; ACE inhibitor: angiotensin converting enzyme inhibitors; SOD: superoxide dismutase; Hsp90: heat shock proteins 90; PI3K/Akt: phosphoinositide-3-kinase–protein kinase; PKM2: M2 isoform of pyruvate kinase; ETA receptor: endothelin-A receptor; CMD: coronary microvascular dysfunction; ROS: reactive oxygen species; RISK/SAPK pathway: extracellular regulated kinase (ERK), protein kinase A (Akt) and eNOS/p38 proteins, and c-Jun N-terminal kinase (JNK); ROS/p-JNK/Bcl-2 pathway: reactive oxygen species/stress-activated c-Jun N-terminal kinase/B-cell lymphoma.
